# Surviving meningococcal septic shock in childhood: long-term overall outcome and the effect on health-related quality of life

**DOI:** 10.1186/cc9087

**Published:** 2010-06-29

**Authors:** Corinne MP Buysse, Lindy CAC Vermunt, Hein Raat, Jan A Hazelzet, Wim CJ Hop, Elisabeth MWJ Utens, Koen FM Joosten

**Affiliations:** 1 Department of Paediatrics, Division of Paediatric Intensive Care, Erasmus MC-Sophia Children's Hospital, Dr. Molewaterplein 60, Rotterdam, 3015 GJ, The Netherlands; 2 Department of Child and Adolescent Psychiatry, Erasmus MC-Sophia Children's Hospital, Dr. Molewaterplein 60, Rotterdam, 3015 GJ, The Netherlands; 3 Department of Public Health, Erasmus MC, Dr. Molewaterplein 60, Rotterdam, 3015 GJ, The Netherlands; 4 Department of Epidemiology and Biostatistics, Erasmus MC, Dr. Molewaterplein 60, Rotterdam, 3015 GJ, The Netherlands

## Abstract

**Introduction:**

The purpose of this study was to evaluate associations between long-term physical and psychological outcome variables in patients who survived meningococcal septic shock (MSS) in childhood.

**Methods:**

The study population was made up of all MSS patients requiring intensive care treatment between 1988 and 2001.

**Results:**

A total of 120 patients visited the follow-up clinic (age at paediatric intensive care unit (PICU) admission 3.1 years; follow-up interval 9.8 years; age at follow-up 14.5 years (all medians)). Four major outcomes were considered: 1) major physical sequelae (defined as major scars and/or orthopaedic sequelae) (29/120), 2) mild neurological impairments (39/120), 3) problem behaviour (defined as a total score above the 90^th ^percentile of the reference groups on questionnaires to screen for psychopathology) (16/114) and 4) total intelligence quotient < 85 (18/115). No differences were found between patients with major physical sequelae and patients without major physical sequelae as to the presence of problem behaviour or total IQ < 85. Also, no differences were found between patients with mild neurological impairments and patients without as to the presence of problem behaviour or total IQ < 85. Finally, no differences were found between patients with major physical sequelae and patients without as to the presence of mild neurological sequelae. Less favourable scores on behavioural and emotional problems were significantly associated with poorer health-related quality of life (HR-QoL). HR-QoL scores were to a lesser amount predicted by severity of illness at time of PICU admission or by adverse physical outcome.

**Conclusions:**

Long-term adverse physical and psychological outcomes in survivors of MSS did not seem to be associated. Poorer HR-QoL was mainly predicted by problem behaviour.

## Introduction

The research presented here is part of a medical and psychological follow-up study of all consecutive surviving patients with meningococcal septic shock (MSS) requiring intensive care treatment between 1988 and 2001 at the Erasmus MC-Sophia Children's Hospital, Rotterdam, The Netherlands. This follow-up study revealed that these patients suffered from mild to severe long-term skin scarring, orthopaedic sequelae and neurological impairments. These patients had significantly higher severity of illness scores than patients without sequelae. Furthermore, they were assigned significantly poorer HR-QoL (health-related quality of life) scores, compared with normative data, though mainly on the physical domains. Overall, behavioural/emotional outcomes and cognitive functioning were normal [1-5].

Until now associations between different outcome variables in MSS survivors have not been investigated. We hypothesize that adverse outcome variables are clustered together, not independently distributed. So patients suffering from severe skin scarring or extensive amputation would also show more problem behaviour and cognitive dysfunctioning. Furthermore we hypothesize that they show poorer HR-QoL.

The present study is by no means a replicate of data already published. Indeed there are points of similarity (the patient sample, outcome variables) among previously published papers in our present study. However, in the present study our primary aim was to evaluate associations between long-term outcome variables, both physical and psychological, in patients who survived MSS in childhood. This knowledge would enable us to identify risk for overall adverse long-term outcome. The secondary aim was to assess various putative determinants of adverse overall outcome and HR-QoL.

## Materials and methods

### Patient selection

Patients were recruited from the PICU of the Erasmus MC-Sophia Children's Hospital, a tertiary care university hospital. Eligible for inclusion were all consecutive surviving patients aged 1 month to 18 years with a clinical picture of MSS, as well as their parents. Meningococcal septic shock was defined as septic shock with petechiae and/or purpura [6]. The Erasmus MC Medical Ethical Review Board approved the study protocol. Written informed consent was obtained from parents and patients after they had received a standard letter requesting their participation. Those with insufficient command of the Dutch language were excluded. Parents and patients who agreed to participate (n = 120) were invited by mail to arrange a visit to the follow-up clinic. The follow-up visits took place in 2005 and 2006.

### Data analysis at PICU admission

During the study period patients consecutively admitted with MSS were included in several sepsis studies [7-11]. Severity of illness was determined by the Pediatric Risk of Mortality Score (PRISM), the Vasopressor score (VAS) and the Disseminated Intravascular Coagulation score (DIC) [12-14].

### Long-term outcome variables

#### Physical health status

Parents and patients were interviewed by one paediatrician (CB) in a semi-structured format using a standard questionnaire with regard to health consequences since MSS. Complaints were defined as chronic if they developed after MSS and were still present at the time of the visit to the follow-up clinic. The same paediatrician physically examined the patients.

Fifty-eight of the 120 patients (48%) had skin scarring due to purpura (ranging from barely visible scars to extremely mutilating scars); 10 (8%) amputation(s) of extremities (ranging from one toe to both legs and one arm); 7 (6%) lower limb-length discrepancy; 42 (35%) neurological impairment(s) (mental retardation with epilepsy, hearing loss, chronic headache or focal neurological signs); and 1(6%) of the 16 patients with septic shock-associated acute renal failure at PICU admission showed signs of mild chronic renal failure [1,3].

#### Psychological functioning

Patients were interviewed and examined by one psychologist (LV) using standard assessment procedures. Intellectual functioning was assessed with two tests for different age ranges: the Wechsler Intelligence Test III (WISC III) for the 6 to 15-year-olds; the Groninger Intelligence Test 2 (GIT2) for the 16 to 31-year-olds [15-17]. Overall, total scores of intellectual functioning were comparable to those of the reference groups [4].

Behavioural and emotional problems were assessed with the Child Behaviour Checklist (CBCL) for the 6 to 18-year-olds, completed by the parents (mothers' reports n = 75, fathers' reports n = 2) and with the Adult Self-Report for the 18 to 31-year-olds patients was used (ASR, n = 37) [18,19]. Overall, no significant differences were found between the proportions of patients (6 to 18 years and 18 to 31 years separately) scoring in the deviant psychopathological range for problem behaviour and same-aged reference groups (adult patients; unpublished data) [5]. The 90th percentiles of the cumulative frequency distributions of the CBCL and ASR total problem scores obtained for the reference groups served as the cut-offs to distinguish patients scoring in the deviant range from non-problem patients.

Scores in the deviant range reflect levels of problem behaviors similar to those of children, adolescents and young adults typically referred for mental health services. Problem behaviour (a dichotomous variable) was defined as a total problem score above the 90^th ^percentile of the cumulative frequency distribution of the reference groups on a) the CBCL or b) the ASR. Youth Self-Report (YSR) ratings of patients aged 11 to 17 years also served to assess behavioural and emotional problems [18].

#### HR-QoL

HR-QoL of patients < 18 years was assessed with the Child Health Questionnaire (CHQ); that of patients ≥ 18 years with the Short-Form 36 [20-24]. Significantly poorer scores were found mainly on physical domains. In patients < 18 years, parents (mothers' reports n = 60, fathers' reports n = 20) assigned significantly poorer scores on psychosocial HR-QoL domains, whereas patients ≥ 12 years self-reported significantly better scores on psychosocial domains [2].

### Statistical methods

Statistical analysis was performed with SPSS 12.0 for Windows (SPSS, Inc, Chicago, IL, USA).

#### Patient sample

Comparisons between participating patients and non-participants were made with the Mann-Whitney test for age at time of PICU admission, length of stay in PICU and severity of illness scores; with the Chi-Square test for sex.

#### Overall physical and psychological outcome

We dichotomised and coded outcome variables (presence or absence of outcome variable), and then generated four major outcome variables; 1) major physical sequelae (n = 29/120) defined as major scars and/or amputation of extremities and/or limb-length discrepancy; 2) mild neurological impairments (n = 39/120) defined as hearing loss and/or chronic headache and/or focal neurological signs; 3) problem behaviour (n = 16/114); and 4) total IQ < 85 (n = 18/115). The latter category included three patients with mental retardation and epilepsy whose intelligence score was estimated to be < 70.

Associations between these major outcomes (all as dichotomous variables) were evaluated with the Chi-Square test.

The psychological outcome variables were also used as continuous variables. In that case the Mann-Whitney test was used to compare these psychological outcome scores between patients with and without major physical sequelae, as well as patients with and without neurological impairments.

#### Predictors of adverse overall outcome

Adverse overall outcome was defined as adverse outcome on one or more of the four major outcome variables. The Mann-Whitney test was used to compare age at the time of PICU admission, length of stay in PICU and severity of illness scores between patients with and without adverse overall outcome.

#### Predictors of HR-QoL

We tested the association between putative predictor variables (patient's characteristics at the time of PICU admission, long-term physical and psychological outcome variables) and long-term HR-QoL scores by using Spearman correlation for continuous variables and Mann-Whitney test for dichotomous variables. This was only done for those HR-QoL scales for which there were significant differences (poorer or better scores) between the study population and the normative data.

In all of the above mentioned statistical analyses, a *P*-value of 0.05 (two-sided) was considered the limit of significance.

Multiple linear regression analyses were applied to evaluate the predictive value of patient characteristics at the time of PICU admission on long-term HR-QoL scores. This was only done for HR-QoL scales if there were significant differences between the study population and the normative data.

In the regression analysis, we included patient characteristics (age at the time of PICU admission, sex), disease variables (severity of illness scores, length of stay in PICU) and follow-up interval. *P*-values of predictors were set to a level of 0.1 in the univariate analysis for entry in the regression analysis. Using backward elimination, independent predictors were identified with a *P*-value < 0.05. Continuous predictors with negative regression coefficients were considered as negatively associated with HR-QoL scales, those with positive values as positively associated.

## Results

### Patient sample

The target population consisted of 179 patients. Nine were lost to follow-up: one patient with severe adverse outcome (mental retardation with epilepsy) died several years after the MSS; seven had moved abroad; one was untraceable. Of the remaining 170 eligible patients, 145 agreed to participate. The other 25 patients and/or parents did not respond to the invitation or refused participation on practical or emotional grounds. Eventually, 120 patients visited the follow-up clinic. The median follow-up interval was 9.8 years (range 3.7 to 17.4 years), the median age of patients at the time of visit to follow-up clinic 14.5 years (range 5.3 to 31.1 years). Twenty-five patients and/or parents did not want to visit the follow-up clinic on practical (for example, no time because of a busy job) or emotional (too emotional confrontation with the hospital) grounds and preferred to fill in the questionnaires at home. The overall response rate, excluding patients lost to follow-up, was 71% (120/170). To check for possible selection bias, we compared characteristics of participants and non-participants (Table 1). Patients did not differ with respect to age at the time of PICU admission and severity of illness.

**Table 1:** Data of participating patients and non-participants. Data are presented as number of patients or median (range)

Characteristics	Follow-up clinic n = 120	No follow-up clinic n = 59	*P*-value
Sex	63 boys, 57 girls	27 boys, 32 girls	ns
Age at admission (years)	3.1 (0.1 to 17.9)	5.4 (0.2 to 14.3)	ns
Length of PICU stay (days)	3 (1 to 51)	3 (1 to 36)	ns
PRISM	15 (1 to 37)	15 (0 to 41)	ns
DIC*	6 (3 to 8)	6 (2 to 8)	ns
VAS	15 (0 to 403)	11 (0 to 145)	ns

*DIC, score ≥ 5 indicates presence of disseminated intravascular coagulation.

PICU, paediatric intensive care unit; PRISM, Pediatric Risk of Mortality Score; VAS, Vasopressor score; DIC, Disseminated Intravascular Coagulation score.

At PICU admission a causative organism was isolated in 100 of the 120 patients (83%) who visited the follow-up clinic. In 99 patients (83%) *Neisseria meningitidis *was cultured in blood. Seventy-eight of these (79%) had NM serogroup B, 13 (13%) serogroup C and in 8 (8%) the serogroup was not determined.

### Overall physical and psychological outcome

Seventy-three of the 120 patients (61%) had adverse outcome on one or more of the four major outcome variables. Forty-seven of these 73 patients had adverse outcome on one major outcome variable: major physical sequelae n = 13, mild neurological impairments n = 19, problem behaviour n = 7 and total IQ < 85 n = 8.

Twenty-six of these 73 patients had adverse outcome on two or three major outcome variables: major physical sequelae *and *mild neurological impairments n = 8, major physical sequelae *and *problem behaviour n = 2, major physical sequelae *and *total IQ < 85 n = 4, mild neurological impairments *and *problem behaviour n = 4, mild neurological impairments *and *total IQ < 85 n = 5, major physical sequelae, mild neurological impairments *and *problem behaviour n = 2, mild neurological impairments, problem behaviour *and *total IQ < 85 n = 1.

The patient with chronic renal failure had amputation of a leg (below-knee), major scars and focal neurological signs. One of the three patients with mental retardation (estimated IQ < 70) had major scars and amputations; another had major scars and lower limb-discrepancy of 13 centimeters.

There were no significant associations between the presence of the four major outcome variables. No differences were found between patients with major physical sequelae and patients without major physical sequelae as to the presence of problem behaviour or total IQ < 85. Also, no differences were found between patients with mild neurological impairments and patients without as to the presence of problem behaviour or total IQ < 85. Finally, no differences were found between patients with major physical sequelae and patients without as to the presence of mild neurological sequelae.

### Predictors of overall physical and psychological outcome

The 73 patients with adverse outcome had significantly longer length of stay in PICU (*P *= 0.003, 4 versus 2 days) and higher severity of illness scores (PRISM *P *= 0.001, 17 versus 12) (VAS *P *= 0.002, 25 versus 6) (all medians) compared with the 47 patients without adverse outcome.

### Predictors of HR-QoL

Univariate analysis of HR-QoL in relation to predictor variables at the time of PICU admission revealed a significant relationship on four HR-QoL scales (Table 2). Age at the time of PICU admission and PRISM showed no significant associations with HR-QoL scores. Multiple linear regression analyses of patient's characteristics at time of PICU admission revealed no significant associations with HR-QoL scores.

**Table 2:** Univariate relations between predictor variables at the time of PICU admission and HR-QoL scales

Predictor variables	Physical functioning	Self-esteem	Family activities	Physical summary (≥ 18 years)
Age at admission	-	-	-	-
PRISM	-	-	-	-
DIC	-	-	0.48**	0.39*
VAS	-	0.26*	-	-
Length of stay in PICU	-.31**	-	-	-

*P *< 0.05 (*), *P *< 0.01 (**), - = ns.

Higher HR-QoL scores indicate more favourable HR-QoL.

The Spearman correlation coefficient is shown. Plus versus minus sign indicates respectively the positive versus negative association between the predictor variable and the HR-QoL scale.

The scales *Physical functioning *and *Self-esteem *are part of the CHQ-PF50 (parent-reports, in patients 4 to 17 years, n = 80).

The scale *Family activities *is part of the CHQ-CF87 (patient-reports, in patients 12 to 17 years, n = 35).

The scale *Physical summary (≥ 18 years) *is part of the SF-36 (patient-reports, in patients ≥ 18 years, n = 38).

Concerning the physical and psychological outcome variables, HR-QoL scores were mainly related with problem behaviour (Tables 3 and 4). There were significant negative associations between all five HR-QoL scales and problem behaviour in the 4-to 17-year-olds (assessed by parents on the CBCL)) (Table 3). Likewise, there were significant negative associations between HR-QoL scales and problem behaviour in those over 12 years of age (total YSR and total ASR) (Table 4).

**Table 3:** Univariate relations between physical and psychological outcome variables and HR-QoL parent-reports

	Physical functioning	General health perception	Self-esteem	Role functioning emotional/behavior	Physical summary
Major physical sequelae	- 6.3*	-	-	-	-
Mild neurological impairments	-	-	-	-	-
IQ < 85	-	-	-	-28.9**	-
Total CBCL^#^	-.25*	-.36**	-.34**	-.33**	-.25*

For dichotomous variables (first three items) the difference (item present minus absent) in mean HR-QoL scale value is shown, for continuous variables (last item) the Spearman correlation coefficient is shown. Plus versus minus sign indicates respectively the positive versus negative association between the predictor variable and the HR-QoL scale.

*P *< 0.05 (*), *P *< 0.01 (**), - = ns.

^#^CBCL (Child Behavior Checklist) is the parent-report of behavioural and emotional problems in patients < 18 years. Higher CBCL scores indicate unfavourable outcome.

The HR-QoL scales are part of the CHQ-PF50 (parent-reports, in patients 4 to 17 years, n = 80).

HR-QoL scales range from 0 to 100. Higher HR-QoL scores indicate more favourable HR-QoL.

**Table 4:** Univariate relations between physical and psychological outcome variables and HR-QoL patient-reports

Predictor variables	Family activities	General health perception	General behaviour	Role limitations emotional	Vitality	Psychosocial summary
Major physical sequelae	6.2*	-	-	-	-	-
Mild neurological impairments	-12.3*	-	-	-10.1*	-11.6*	
Total YSR^#^	-.52**	-.34*	-.60**	-	-	-
Total ASR^##^	-	-	-	-.53**	-.44**	-.56**

For dichotomous variables (first two items) the difference (item present minus absent) in mean HR-QoL scale value is shown, for continuous variables (last two items) the Spearman correlation coefficient is shown. Plus versus minus sign indicates respectively the positive versus negative association between the predictor variable and the HR-QoL scale.

*P *< 0.05 (*), *P *< 0.01 (**), - = ns.

^# ^YSR (Youth Self-report) and ASR ^## ^(Adult Self-Report) are self-reports of behavioural and emotional problems in resp. patients 11 to 17 years and > 18 years. Higher YSR and ASR scores indicate unfavourable outcome.

The scales *Family activities*, *General health perception*, *General behaviour *are part of the CHQ-CF87 (patient-reports, in patients 12 to 17 years, n = 35).

The scales *Role limitations due to emotional problems*, *Vitality*, *Psychosocial summary *are part of the SF-36 (patient-reports, in patients ≥ 18 years, n = 38).

HR-QoL scales range from 0 to 100. Higher HR-QoL scores indicate more favourable HR-QoL.

## Discussion

From the results of this study we may conclude that major physical sequelae and mild neurological impairments in these survivors of childhood MSS are not associated with problem behaviour or total IQ < 85. Furthermore, problem behaviour was significantly associated with poorer HR-QoL.

### Overall outcome

To the best of our knowledge, this is the first study investigating associations between different long-term outcome variables in MSS. Erickson *et al. *described that "some patients had multiple sequelae" [25]. Fellick *et al. *classified the level of impairment in different categories, based on physical outcome, total intelligence and motor skills [26]. These authors, however, did not evaluate correlations between different outcome variables. In our homogeneous patient sample of MSS survivors those with major physical sequelae after MSS had no more neurological impairments, nor more cognitive dysfunctioning or problem behaviour than those without major physical sequelae. Several explanations present themselves. For one, after a life-threatening illness such as MSS, one may have greater appreciation of life. Indeed, many parents and patients reported that the event had made them stronger and that they tried to make the best of their lives. This phenomenon is referred to as *resilience *in our previous study [5]. This resilience may be stronger than that in adult patients with critical illness, as suggested by Erickson *et al. *[27]. These authors reported a great deal of "emotionally unresolved grief" (anger, anxiety, depression) in adults who survived invasive meningococcal disease, several years after hospital discharge.

Second, the major physical sequelae were directly related to the severity of MSS [3]. Severity of illness scores, however, were not significant predictors of long-term mild neurological impairments, levels of behavioural problems nor of cognitive dysfunction [1,4,5]. It would seem that these latter outcomes were not specifically related to shock and intravascular thrombosis, but rather to acute illness in general. Furthermore, total scores of intellectual functioning and of behavioural and emotional problems in our study group were comparable to those of the reference groups.

We like to add, as is described in details elsewhere, that no association was found between putative predictor variables (age at time of PICU admission, severity of illness scores, presence of meningitis, convulsions and corticosteroids therapy during PICU admission) and long-term mild neurological impairments [1]. As to the association of raised intracranial pressure and long-term mild neurological impairments, measurement of intracranial pressure was not indicated (and was even contraindicated) in our study group, that is, patients with a clinical picture of meningococcal septic shock. Indeed most children had symptoms like altered consciousness and vomiting. However these are nonspecific symptoms of both meningococcal septic shock and elevated intracranial pressure. It could be interesting to test the association between the presence of delirium and adverse neurological outcome. Unfortunately, the presence of delirium after PICU admission was not investigated.

So in our study group it seems that adverse long-term physical and psychological outcomes in survivors of MSS were not related. However, some specific cases should be mentioned, but they were insufficient to result in statistically significant differences: for example, two of the three patients with mental retardation (estimated IQ < 70) had major physical sequelae; the patient with chronic renal failure had major physical sequelae and focal neurological signs.

### Predictors of HR-QoL

Long-term poorer HR-QoL was mainly predicted by less favourable scores on behavioural and emotional problem scales. HR-QoL scores, both on the physical and psychosocial domains, were significantly associated with problem behavior (total CBCL, total YSR, total ASR), regardless of age and parent-report versus patient-report. These findings are in line with those from a study by Koomen *et al. *in children 4 to 10 years after bacterial meningitis [28]. This comparison requires caution, however, because MSS is a more severe disease (for example, multiple organ failure) than bacterial meningitis.

HR-QoL scores were to a lesser amount significantly associated with adverse physical outcome; patients with major physical sequelae had significantly poorer scores on the HR-QoL scale *physical functioning*. Indeed, amputation or limb-length discrepancy often resulted in important long-term morbidity (pain, functional impairment) in our study group [3]. Surprisingly the HR-QoL scale *general health perception *was not significantly associated with the presence of major physical sequelae or mild neurological impairments. The significantly poorer scores on this HR-QoL scale, therefore, most likely reflected future health status, rather than the present one.

Studies regarding long-term HR-QoL in survivors of other severe illnesses, for example, congenital heart diseases, also demonstrated weak associations between present physical health status and HR-QoL [29,30].

Seeing that DIC and VAS, but not PRISM, showed small to moderate correlations (r_s_< 0.7) on a minority of HR-QoL scales only in univariate analysis, the severity of MSS in childhood, regardless of any adverse outcome, seemed less important for long-term HR-QoL.

### Limitations of the present study

Several limitations of our study should be acknowledged. This is an observational study (without a suitable control group) in one centre. The response rate was not high (71%), but we believe the results are valid since participating patients and non-participants did not differ with respect to age at time of PICU admission and severity of illness. It should be emphasized that only the most critically ill patients, that is, MSS patients requiring intensive treatment, were included. Therefore our findings cannot be extrapolated to the milder cases (sepsis) admitted to a general ward. Finally, baseline assessments of health status, psychological functioning and HR-QoL (before MSS) were not available, which would obviously be difficult to measure reliably under such stressful circumstances.

## Conclusions

Long-term adverse physical and psychological outcome variables were independently distributed, not clustered together. For example, patients suffering from severe skin scarring or extensive amputation did not show more problem behaviour and cognitive dysfunctioning compared with patients without severe skin scarring or extensive amputation. Poorer HR-QoL was mainly predicted by problem behaviour.

More conclusive evidence could be obtained from a study comparing MSS survivors with survivors of other critical illness, matched on age and follow-up interval.

## Key messages

• Seventy-three of the 120 patients (61%) had adverse outcome on one or more of the physical and psychological outcome variables.

• The 73 patients with adverse outcomes had significantly longer length stays in PICU and higher severity of illness scores compared with the 47 patients without adverse outcomes.

• Patients suffering from severe skin scarring or extensive amputation did not show more problem behaviour and cognitive dysfunction compared with patients without severe skin scarring or extensive amputation.

• Patient's characteristics at the time of PICU admission revealed no significant associations with HR-QoL scores.

• Concerning the physical and psychological outcome variables, HR-QoL scores were mainly related with problem behaviour.

## Abbreviations

ASR: Adult Self-Report; DIC, CBCL: Child Behaviour Checklist; CHQ: Child Health Questionnaire; Disseminated Intravascular Coagulation score; HR-QoL: health-related quality of life; MSS: meningococcal septic shock; NM: *Neisseria meningitidis*; PICU: paediatric intensive care unit; PRISM: Pediatric Risk of Mortality Score; VAS: vasopressor score; YSR: Youth Self-Report.

## Competing interests

The authors declare that they have no competing interests.

## Authors' contributions

CMPB initiated this study and created the database, performed the statistical analysis and wrote the manuscript. LCACV assisted in creating the database, the interpretation of the results and writing of the manuscript. HR assisted in interpretation of the results and critically read the manuscript; JAH critically read the manuscript and assisted in interpretation of the results. WCJH performed the statistical analysis and assisted in the interpretation of the results and writing of the manuscript. EMWJ assisted in the interpretation of the results and writing of the manuscript. KFMJ initiated this study and assisted in the interpretation of the results and writing of the manuscript.
